# Optimization of Isavuconazole Dosing in Patients with Invasive Fungal Infections Through Therapeutic Drug Monitoring: Real-World Clinical Practice Experience

**DOI:** 10.3390/life15060946

**Published:** 2025-06-12

**Authors:** Diego Peña-Lorenzo, Noemí Rebollo, José Germán Sánchez-Hernández, Lourdes Vázquez-López, María José Otero, Aránzazu Zarzuelo-Castañeda

**Affiliations:** 1 Pharmacy Service, University Hospital of Salamanca, 37007 Salamanca, Spainmjotero@saludcastillayleon.es (M.J.O.); 2 Biomedical Research Institute of Salamanca (IBSAL), 37007 Salamanca, Spain; 3 Haematology Service, University Hospital of Salamanca, 37007 Salamanca, Spain; 4 Department of Pharmaceutical Sciences, Faculty of Pharmacy, University of Salamanca, 37007 Salamanca, Spain

**Keywords:** isavuconazole, therapeutic drug monitoring, invasive fungal infection

## Abstract

Therapeutic drug monitoring (TDM) is routinely recommended for most antifungal triazoles to ensure efficacy and safety. Isavuconazole, however, was initially approved without this recommendation due to its predictable pharmacokinetic profile. Later clinical data have raised concerns about subtherapeutic exposures in certain populations. This prospective, single-center study aimed to assess the need for TDM of isavuconazole in critically ill and hematologic patients with invasive fungal infections. Between March 2022 and November 2023, patients receiving standard dosing of isavuconazole were enrolled, and plasma concentrations were measured to determine the proportion of patients with values outside the therapeutic range (1–4 µg/mL), particularly focusing on subtherapeutic levels. A total of 65 isavuconazole plasma concentrations from 24 patients (9 critically ill and 15 hematologic) were analyzed. Critically ill patients had lower initial concentrations than hematologic patients (median [range]: 0.75 [not detectable (ND)–5.18] vs. 3.03 [1.03–6.65] µg/mL), with 66.7% showing levels outside the therapeutic range and 55.5% having subtherapeutic concentrations. The coefficient of variation (CV%) of concentrations values at the first TDM was 124.7% in critically ill patients and 57.3% in hematologic patients. After dose adjustment in critically ill patients, the proportion with levels outside the therapeutic range decreased to 28.6%. These findings suggest that, despite initial assumptions, isavuconazole exhibits considerable pharmacokinetic variability in specific populations, particularly in critically ill patients, and the findings support the implementation of TDM to optimize antifungal therapy and improve patient outcomes in real-world clinical settings.

## 1. Introduction

Invasive fungal infections (IFIs) represent a significant challenge for clinicians, given that the number of patients susceptible to developing these infections has increased considerably in recent years, and that they are associated with a high mortality rate [1]. Triazoles are first-line antifungal agents for the treatment and prophylaxis of IFIs. Isavuconazole is the latest triazole commercialized, being the only one approved by EMA and FDA for the treatment of both invasive aspergillosis and mucormycosis [2]. In comparison with other azoles, isavuconazole has a more favorable safety profile. While azoles are known to prolong the QTc interval, clinical trials have shown that isavuconazole administration may cause a dose-dependent QTc shortening [3]. Moreover, since it is only a moderate inhibitor of CYP3A4, fewer relevant drug–drug interactions than other antifungal triazoles have been described [2,4].

Therapeutic drug monitoring (TDM) is a tool routinely recommended to optimize the safety and efficacy of treatment with itraconazole, voriconazole, and posaconazole, due to their significant pharmacokinetic variability and number of drug interactions [4]. However, when isavuconazole was approved, TDM was not considered necessary for this drug, given its lower potential for interactions and more predictable pharmacokinetic profile, as shown in the pivotal VITAL and SECURE clinical trials, which demonstrated minimal intra- and inter-individual variability in its plasma concentrations [5,6].

Although the results obtained in the pivotal clinical trials did not justify the need for TDM, some authors have reported concentrations not included within the expected range. This phenomenon could not be explained by any known factor affecting the isavuconazole PK, such as drug interactions or CYP3A4/5 polymorphisms [4]. Furthermore, subsequent studies in real-life practice have reported plasma concentrations that differ from expectations in certain patient groups, such as those undergoing renal replacement therapy, extracorporeal membrane oxygenation (ECMO), with moderate hepatic impairment, obesity, as well as in pediatric populations [2,7,8,9].

Moreover, several population pharmacokinetic models of isavuconazole have been developed to support individualized dosing and TDM. Early models were based on clinical trial data (e.g., Kovanda et al., Desai et al.) [5,10], while more recent models have been derived from real-world patient cohorts (e.g., Cojutti et al.) [11], in response to the greater pharmacokinetic variability observed in clinical practice. These models aim to better characterize isavuconazole behavior across diverse patient populations. This information, along with studies that proposed a minimum effective concentration of >1 µg/mL and a toxicity threshold of 4.6–5.1 µg/mL [12,13,14], has led to a reassessment of the utility of isavuconazole TDM in specific patients to ensure that effective plasma concentrations are achieved [15].

The aim of this study was to determine whether or not isavuconazole TDM is necessary to optimize dosing in critically ill and hematologic patients with invasive fungal infections treated at our center.

## 2. Materials and Methods

### 2.1. Study Design and Population

This was a single-center, prospective, non-controlled interventional study conducted at a fourth-level hospital. The study protocol, which included the implementation of TDM for various triazoles in patients with IFIs, was approved by the hospital’s Clinical Research Ethics Committee (Ethics Committee number: PI2020/03/460).

The study included critically ill adult patients admitted to intensive care, as well as hospitalized or outpatient hematologic patients who received intravenous or oral isavuconazole treatment for IFIs. Plasma concentrations were monitored between March 2022 and November 2023.

Patients initially received the standard isavuconazole dosing regimen according to the Summary of Product Characteristics: a loading dose of 200 mg every 8 h for 48 h, followed by a maintenance dose of 200 mg every 24 h. Isavuconazole concentrations were measured as part of routine clinical care at steady state, just prior to each intravenous or oral dose (C_min_). Steady state was considered to be reached after completion of the loading phase and prior to the first maintenance dose, for both intravenous and oral administration. In hospitalized patients, samples were obtained at steady state, immediately before intravenous or oral administration. In outpatients, samples were also obtained at steady state, within a maximum of 4 h prior to oral administration, due to the minimal fluctuation in concentration resulting from isavuconazole long elimination half-life.

For each patient, the following information was recorded: sex, age, height, weight, body mass index (BMI), total bilirubin, aspartate aminotransferase (AST), alanine aminotransferase (ALT), alkaline phosphatase, gamma-glutamyl transferase (GGT), route of administration, number of treatment days until monitoring, and isavuconazole concentration.

### 2.2. Isavuconazole Determination

Isavuconazole pure drug substance was provided by the Sigma-Aldrich^®^ laboratory (St. Louis, MO, USA). HPLC grade acetonitrile was supplied by Thermo Fisher Scientific^®^ (Waltham, MA, USA). Formic acid was purchased from the Sigma-Aldrich^®^ laboratory. Ultrapure water was obtained with a Wasserlab Automatic Plus System (West Valley, UT, USA).

Plasma concentrations of isavuconazole were measured by reverse-phase ultra-performance liquid chromatography (UPLC)) on Waters Acquity High Pressure Chromatographer, using a Luna Omega C18 column (1.6 μm; 2.1 mm × 50 mm, Phenomenex Company (Torrance, CA, USA)). The mobile phase used for the elution consisted of a 55/45 mixture of 0.5% formic acid and acetonitrile; the flow rate was 0.5 mL/min and the UV detector was set at 285 nm. The column temperature was set at 40 °C, the sample manager was operated at 20 °C, and the sample injection was 10 µL in a full-loop model.

A stock solution of isavuconazole (100 µg/mL) was prepared in acetonitrile. Calibration standards and quality control (QC) samples were prepared by diluting this stock solution in blank human plasma. Final concentrations of the calibration standards were 0.25, 0.5, 1.0, 2.5, 5.0, 7.5, 10.0, 12.5, and 15.0 µg/mL, and the concentrations of the QC samples were 0.25, 1.0, 5.0, and 12.5 µg/mL. All stock solutions, calibration standards, and QCs were stored at −20 °C until use.

Before chromatographic injection, the samples were treated by adding 100 µL of acetonitrile to 100 µL of plasma; after vortexing for 30 s, the sample was centrifuged at 5000 rpm for 5 min. The resulting supernatant was filtered through a 0.22 µm PVDF syringe filter (Millex^®^, MilliporeSigma, Burlington, MA, USA), and injected into the UPLC system. No internal standard was used, as the method relies on direct protein precipitation followed by UV detection. The extraction efficiency of the direct plasma deproteinization method, verified in previous internal validations, was 92, 97, 104 and 102% at 0.25, 1.0, 5.0, and 12.5 µg/mL of isavuconazole, respectively, against the water.

The method was adequately validated for specificity, linearity, precision, accuracy, carry over, and stability according to FDA Guidance and EMA Guidelines on bioanalytical method validation [16,17]. The calibration range of the assay was 0.25–15.0 μg/mL. The lower limit of quantification (LLOQ) in human plasma was 0.25 µg/mL, with a relative standard deviation (RSD) of 8.33% and a relative error (RE) of −8.11%, confirming acceptable precision and accuracy at this level.

### 2.3. Isavuconazole Dose Individualization

The optimal dose for each patient was estimated using a Bayesian method. Nonlinear mixed-effects models (NONMEM version 7.4.0) were applied utilizing the population pharmacokinetic model proposed by Cojutti et al. [11]. This model was selected because it was developed using real-world clinical data from patients with invasive fungal infections—particularly hematologic and immunocompromised individuals—closely resembling much of our target population. Although critically ill patients were not specifically included in the original Cojutti cohort, the model was suitable for Bayesian dose individualization in our diverse clinical setting. A therapeutic range of 1–4 µg/mL for isavuconazole plasma concentrations was established [14,15]. After each monitoring, a report was generated including the recommended dose for the patient and the suggested timing for repeat monitoring if a significant dose adjustment was advised or if clinical conditions warranted it. This recommendation was based on model-informed simulations using individual pharmacokinetic parameters estimated via Bayesian forecasting, with the goal of identifying the most appropriate dosing regimen to achieve therapeutic plasma concentrations.

To assess the need for isavuconazole TDM, the percentage of patients with C_min_ values outside the therapeutic range and those below 1 µg/mL were calculated for the initial monitoring after standard dosing, as well as for any subsequent monitoring when applicable. Critically ill and hematologic patients were analyzed separately. Comparisons of isavuconazole C_min_ values between both groups at the first and second TDM were performed using the Mann–Whitney U test. A two-sided *p*-value < 0.05 was considered statistically significant. Statistical analysis was performed using SPSS version 25 (IBM Corp., Armonk, NY, USA).

## 3. Results

A total of 24 patients receiving isavuconazole for IFI were included: 9 critically ill patients receiving intravenous therapy, and 15 hematologic patients (12 hospitalized and 3 outpatients), of whom 11 received oral therapy and 4 intravenous. The baseline characteristics of the patients are summarized in Table 1.

**Figure 1:**
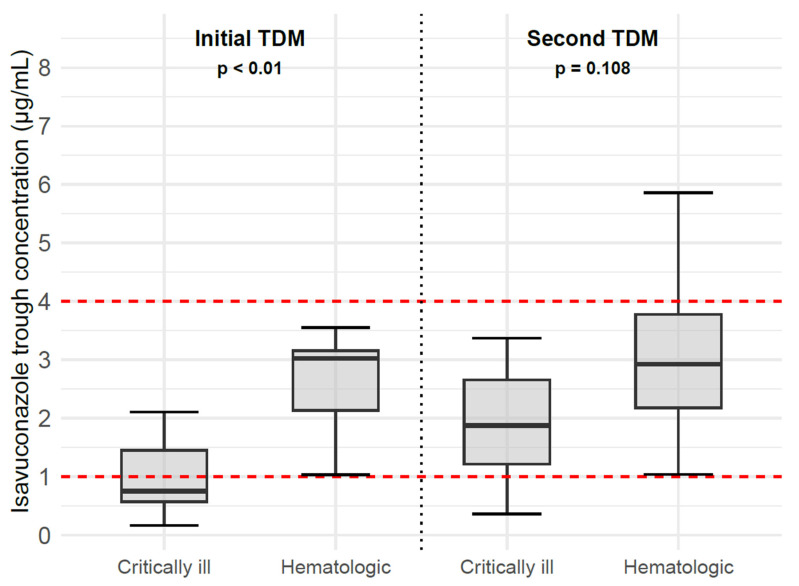
Boxplot of isavuconazole trough concentrations in the first therapeutic drug monitoring (TDM) after administration of the standard dose (left side), and in the second TDM after dose optimization (right side), in critically ill and hematologic patients. Each box represents the interquartile range distribution of isavuconazole trough concentrations. The bold lines inside the boxes indicate the median. The red dashed line represents the therapeutic range of isavuconazole.

A total of 65 isavuconazole plasma concentrations were determined, with a median (range) of 2 (1–6) determinations per patient. Figure 1 graphically illustrates the distribution of isavuconazole C_min_ values in critically ill and hematologic patients at the time of the first measurement and after the first dose adjustment based on TDM. In the first monitoring, the median (range) of isavuconazole C_min_ across all patients was 2.45 (ND–6.65) µg/mL. Initial isavuconazole C_min_ values were lower in critically ill patients compared to hematologic patients, with a median of 0.75 (ND–5.18) µg/mL vs. 3.02 (1.03–6.65) µg/mL (*p* < 0.01). After dose adjustment, in the second monitoring, C_min_ values remained lower in critically ill patients—1.87 (0.36–3.37) µg/mL vs. 2.93 (1.04–5.86) µg/mL—but the difference was not statistically significant (*p* = 0.108). The coefficient of variation (CV%) of C_min_ at the first TDM was 124.7% in critically ill patients and 57.3% in hematologic patients, indicating markedly higher variability in the former. After dose adjustment, CV% decreased to 62.4% in critically ill patients and 49.1% in hematologic patients.

Table 2 presents the main results from the TDM analysis. The percentage of isavuconazole C_min_ values outside the therapeutic range at the first monitoring, following standard dosing, was 33.3% across all patients. Among critically ill patients, this percentage was 66.7%, with subtherapeutic concentrations found in five out of nine patients (55.5%). After dose adjustment based on TDM, the percentage of C_min_ values outside the therapeutic range decreased to 28.6% among critically ill patients. Among hematologic patients, 13.3% (2/15) initially had concentrations outside the defined therapeutic range.

## 4. Discussion

TDM of voriconazole, posaconazole, and itraconazole is essential for optimizing the dosing of these antifungal agents, thereby increasing the likelihood of therapeutic success in patients with IFIs, especially given the serious consequences of inadequate antifungal therapy [4]. However, current evidence regarding the need for routine isavuconazole TDM and the specific patient groups who might benefit from it remain limited.

In our study, 33.3% of patients had isavuconazole C_min_ values outside the therapeutic range during the first TDM, a proportion similar to that observed in other real-world clinical studies. Andes et al. [12], in the United States, found that approximately 35% of patients had C_min_ values outside the therapeutic range, which matched the range used in our study (1–4 µg/mL). Based on these results, they concluded that routine TDM is not necessary. Conversely, Borman et al. [18], in the United Kingdom, used a more restrictive range (2–4 µg/mL) and observed that 58.6% of patients had values outside this range. They recommended TDM particularly for pediatric patients and during prolonged treatment.

Our findings suggest that TDM may be especially useful for critically ill patients. These patients had lower initial isavuconazole concentrations than did hematologic patients—with a median of 0.75 µg/mL—below the therapeutic range. Consequently, 66.7% of critically ill patients had C_min_ levels outside the therapeutic range, and in 55.5% of cases (5/9), the values were subtherapeutic. It is important to note that if a lower threshold of 2 µg/mL were adopted—as suggested by some studies [18,19,20]—the proportion of critically ill patients with subtherapeutic concentrations would rise to 77.8%, further reinforcing the need to consider TDM in this population in order to optimize treatment.

Other recent real-world studies have shown greater variability in isavuconazole plasma concentrations among critically ill patients [21,22]. Low C_min_ values (<1 µg/mL) have been associated with factors such as obesity, age under 65 years, renal replacement therapy, ECMO, and higher SOFA (Sepsis-Related Organ Failure Assessment) scores. These studies support the use of TDM in critically ill patients to improve clinical outcomes.

In haematological patients, although a wide range of plasma concentrations was observed at the second monitoring, the overall coefficient of variation (CV%) decreased from 57.3% to 49.1% following TDM-guided dose adjustments, suggesting improved consistency of drug exposure at the group level. However, significant individual variability remained, potentially reflecting clinical changes (e.g., gastrointestinal complications, hepatic alterations, concomitant therapies) or limitations inherent to the pharmacokinetic model used for dose estimation.

This study has significant limitations, primarily due to its single-center design and the small sample size. Our limited number of patients prevented an in-depth analysis of clinical factors or concomitant therapies that could affect isavuconazole pharmacokinetics. In addition, the relationship between TDM results and treatment efficacy or toxicity was not assessed, mainly due to the challenges of systematic follow-up in routine clinical practice, such as early discharge or inter-hospital transfers. Furthermore, although the pharmacokinetic model used for dose estimation was developed in a real-world cohort and was internally validated with local patient data prior to clinical implementation, it has not been specifically validated in critically ill or haematologic patients within the scope of this study. This may limit its predictive performance in these subgroups. Future studies should formally evaluate its predictive accuracy—using relative and absolute error analyses—to support its application in these populations. Despite these limitations, our data indicate that patients with IFIs—especially critically ill patients—have variable isavuconazole concentrations that are often lower than expected, potentially compromising treatment effectiveness.

In conclusion, a significant proportion of patients—particularly critically ill patients—with IFIs treated with standard isavuconazole doses exhibit plasma concentrations outside the therapeutic range. Isavuconazole TDM emerges as a key tool for personalizing treatment in these severely ill patients, allowing dose optimization to achieve therapeutic plasma levels. Further studies with larger cohorts are needed to better characterize the pharmacokinetics of isavuconazole in critical populations.

## Figures and Tables

**Table 1:** Baseline and treatment characteristics of the patients included in the study.

Characteristics	Total Patients (n = 24)	Critically Ill (n = 9)	Hematologic (n = 15)
Male sex, %	66.7	88.9	53.3
Age (years), median [range]	60.5 [36.0–89.0]	62.0 [53.0–76.0]	58.0 [36.0–89.0]
Height (m), median [range]	1.69 [1.54–1.84]	1.70 [1.64–1.80]	1.68 [1.54–1.84]
Weight (kg), median [range]	77.5 [45.0–150.0]	82.0 [74.0–150.0]	67.8 [45.0–97.0]
BMI (kg/m^2^), median [range]	26.8 [17.6–46.3]	27.6 [25.4–46.3]	24.3 [17.6–30.9]
**Biochemical parameters**, median [range]			
Total bilirubin (mmol/L)	0.71 [0.21–4.76]	0.73 [0.21–3.83]	0.71 [0.29–4.76]
Aspartate aminotransferase (U/L)	41.5 [11.0–568.0]	122.0 [37.0–568.0]	36.0 [11.0–79.0]
Alanine aminotransferase (U/L)	36.0 [5.0–760.0]	81.5 [18.0–767.0]	24.0 [5.0–132.0]
Alkaline phosphatase (U/L)	112 [55–811]	138 [55–811]	92 [58–703]
γ-glutamyl transferase (U/L)	97.0 [8.0–565.0]	200.5 [65.0–565.0]	64.0 [8.0–180.0]
**Isavuconazole therapy** (IV/Oral), n	13/11	9/0	4/11
**Treatment setting** (HOSP/OUT), n	21/3	9/0	12/3

BMI: body mass index; HOSP: hospitalized patients; IV: intravenous; O: oral; OUT: outpatients.

**Table 2:** Isavuconazole C_min_ results in the patients included in the study.

Isavuconazole Concentrations	Total Patients (n = 24)	Critically Ill (n = 9)	Hematologic (n = 15)
**Days of treatment until TDM**, **median [range]**	6.0 [2–49]	7.5 [2–31]	6.0 [3–49]
**Initial TDM**			
Concentration (µg/mL), median [range]	2.45 [ND–6.65]	0.75 [ND–5.18]	3.02 [1.03–6.65]
% of concentrations outside range	33.3	66.7	13.33 (2/15)
% of subtherapeutic concentrations	20.8	55.5	0 (0/15)
**Second TDM**			
Concentration (µg/mL), median [range]	2.60 [0.36–5.86]	1.87 [0.36–3.37]	2.93 [1.04–5.86]
% of concentrations outside range	26.3	28.6	25.0
% of subtherapeutic concentrations	10.5	28.6	0

ND: not detectable; TDM: therapeutic drug monitoring.

## Data Availability

The original contributions presented in this study are included in the article. Further inquiries can be directed to the corresponding author.
